# Cardiovascular evaluation of athletes ahead of participation

**DOI:** 10.3389/fcvm.2025.1666981

**Published:** 2025-10-03

**Authors:** Arijita Banerjee

**Affiliations:** Physiology, IIT Kharagpur, Kharagpur, India

**Keywords:** sports medicine, prescreening, cardiovascular evaluation, electrocardiography (ECG), sudden cardiac arrest (SCA)

## Introduction

Regular exercise training and physical activity are linked to several health benefits, particularly at the cardiovascular (CV) level, enhancing quality of life, lowering the incidence of cardiovascular diseases, and preventing and controlling a number of risk factors (1, 2). Despite the overwhelming evidence of its many positive effects, exercise can cause serious clinical complications, including sudden cardiac death (SCD), in people with certain risk factors and cardiovascular disease (CVD). These complications can affect a wide range of people who appear to be healthy, from young athletes to seasoned athletes (3). Through preparticipation screening (PPS), cardiac disorders and abnormalities in electrophysiology of heart that could cause lethal arrhythmias during exercise can be identified. Athletes should be evaluated on a regular basis because SCD may be the initial sign of a hidden illness in asymptomatic people. The purpose of competitive cardiovascular screening prior to participation is not only to check for underlying cardiovascular abnormalities that could raise their risk of sudden cardiac death (SCD) while participating in sports but also highlights the importance of moving the assessment of CVD risk factors beyond the traditional focus to a more holistic and individualized approach like lifestyle modification, restriction from sports activity, and, when appropriate, medical therapy or implantation of a defibrillator (4–6).

Preparticipation screening is crucial for reducing sports-related complications, but some conditions may not be detectable. To increase survival after sudden cardiac arrest, bystanders must be capable of performing resuscitation of the heart particularly using defibrillators. Time between collapse and defibrillation determines survival (7).

The field of sports cardiology is constantly changing, with new research opportunities and some contentious issues. As in other medical fields, for instance, the use of digital tools, such as machine learning shows promise and may aid in better screening. There may be multiple options available to improve risk assessment, diagnosis, and monitoring of cardiovascular health of an athlete by integrating these tools (8, 9).

### Causes of sudden cardiac death in athletes

According to previous research, the incidence of SCD was 6.64 per 100,000 person-years for older athletes over 35 and 0.47–1.21 per 100,000 person-years for young competitive athletes. In addition to age, other factors like gender, sports tenacity, and level of competition appear to influence the prevalence of SCD in athletes. More than 80% of SCD causes in athletes have a cardiovascular origin, primarily from inherited cardiac conditions in young athletes and coronary artery disease (CAD) in older athletes. Cardiomyopathies continue to be significant causes of inherited conditions, but recent studies have shown an increasing prevalence of cases involving hearts with normal physiology, that indicate sudden arrhythmia leading to death, dysautonomia and primary arrhythmia are the most likely underlying aetiology (10–12).

The significance of autopsy in SCD cases should be emphasised because autopsy imaging is still insufficiently reliable to identify certain cardiac diseases, and death certification by itself can be erroneous. Improved risk stratification and the development of preventative measures for vulnerable population, are crucial because genetic conditions account for a large portion of SCD cases in younger athletes (13). Molecular autopsy could be a crucial step in the assessment of SCD patients. Heat stroke, cardiopulmonary disease, and performance-enhancing substances are examples of environmental factors that are associated with SCD in athletes and should be taken into account (14, 15).

### Various cardiovascular screening techniques for athletes ahead of participation

#### History and clinical evaluation

A thorough 12-lead electrocardiography (ECG), physical examination and medical history should be the main focus of pre-participation screening (PPS) for young athletes. Symptoms like palpitations, dizziness, syncope, or exertional chest pain should be considered warning signs. One crucial piece of advice is to teach athletes not to train when they have a fever or before fully recovering from an acute illness. The main goals of the physical examination are to rule out systolic and diastolic heart murmurs high blood pressure, radiofemoral delay, arrythmia, any musculoskeletal and ocular symptoms that could indicate Marfan syndrome. In many nations, the history and physical examination is still the most effective way to screen athletes, but there are some serious drawbacks. There is a great deal of variation in the providers' capacity to use cardiac auscultation to diagnose cardiac conditions during the physical examination portion of the test (16, 17).

#### Electrocardiography (ECG)

The main evidence in favour of ECG use comes from a small number of prospective studies in which the SCD rate was significantly lowered from 3.6% to 0.4% through systematic ECG screening. The sensitivity and specificity are increased by ECG as opposed to just a clinical history and physical examination. As ECG screening guidelines have evolved, the International Criteria now have significantly lower false positive rates, ranging from 1.3% to 6.8%, depending on the population under study. It is crucial to distinguish between pathological and physiological conditions in order to accurately identify athletes who are more vulnerable. One crucial step in ensuring high quality and lowering false positive results is the analysis and proper application of ECG criteria in athletes. Over the past twenty years, the standards for interpreting an athlete's ECG have been refined and shared (18).

Despite these advancements, physicians still need to receive training in athletes' ECG interpretation. Left ventricular hypertrophy, isolated right bundle branch block, nonpathological T wave inversion (TWI), J waves, and nonpathological rhythm variations are common findings that are misinterpreted. ECG screening opponents frequently point to the high expense of ECGs, high false-positive rates that result in unnecessary secondary testing, a lack of generalised proficiency in interpreting an athlete's ECG, and the incapacity to identify critical conditions like congenital anomalous coronary arteries as reasons why universal screening is not necessary. Other ECG findings, such as ST-segment depression, low QRS voltage, premature beats, premature ventricular contractions (PVC) and QRS fragmentation may also have clinical significance, according to recent evidence. To fully comprehend its clinical utility, more investigation is required (19, 20).

#### Echocardiography

Transthoracic echocardiograms (TTE) can increase the precision of PPS by identifying certain conditions linked to SCD that would go undetected by a standard PPS, such as mitral valve prolapse, aortic bicuspid valve, and abnormal coronary artery origin. There is a fee associated with incorporating TTE into a PPS. It makes the PPS more complicated, places a significant financial strain on society, necessitates more extensive follow-up and initial PPS logistics, is operator dependent, and requires training, expertise, and echocardiography experience (21).

Even though a single TTE performed during adolescence will rule out congenital cardiac conditions, it might not detect cardiomyopathies, which might not manifest phenotypically until later. This point emphasises the necessity of reevaluating these athletes in the future, but it is still unclear when the TTE should be repeated. It should be made clear what the ideal age is for the first PPS, how often it occurs, and whether it should change depending on the sport. There is still a lack of knowledge regarding the role of TTE in athletes' PPS, the best protocol to follow, and when to reevaluate. Apart from FIFA, no significant professional or athletic organisation currently advises routine multimodality imaging as part of cardiovascular screening. However, a number of screening initiatives, academic institutions, national teams, and professional teams have incorporated imaging into their standard screening procedure (22, 23).

#### Exercise testing

For many years, asymptomatic people with elevated cardiovascular risk factors who plan to begin regular exercise training have been screened using exercise testing (ET). Given its accessibility, affordability, and status as one of the important functional investigations, ET is highly valuable for assessing veteran athletes with cardiovascular risk factors as well as sedentary individuals planning to start vigorous exercise. Given the increased incidence of coronary artery disease, CV screening in athletes must focus on this condition. ET's diagnostic precision in asymptomatic people without CV risk factors is questionable, though. Due to susceptible plaques that could burst and cause sudden cardiac arrest during exercise, ET may not detect small stenosis even though it may be sensitive in detecting obstructive CAD (24, 25).

#### Artificial intelligence (Ai) in sports cardiology

Modern imaging and ECG software has completely changed how accurately data is gathered during cardiac evaluations. When it comes to identifying asymptomatic cardiac abnormalities that could be dangerous during sports, these tools are invaluable. At the same time, sports medicine is changing due to the growing popularity of consumer wearable devices (CWDs), like heart rate monitors and activity trackers. CWDs primarily use photoplethysmography (PPG) for physiological measurements, and PPG is especially common in devices worn on the fingers and wrists. Wearable technology has great promise for widespread remote monitoring and early cardiac irregularity detection, improving preventive care by delivering an extensive spectrum of cardiopulmonary metrics straight to consumers. Training regimens and customised exercise recommendations for athletes with cardiac conditions can be created by integrating data from these devices to guide training intensity. Use of machine learning has also been implemented by using the ECG data in generation of heart rate variability (HRV) reports for diagnosing any autonomic dysfunction giving rise to arrythmia (26–31). As the current gold standard for assessing myocardial structure and tissue architecture, cardiac magnetic resonance imaging (CMR) is a well-established imaging modality for assessing the cardiovascular system in athletes. It can be difficult to accurately interpret CMR images, and mistakes can have serious repercussions, even for athletes. Accurate interpretation and increased efficiency are two benefits of integrating ML into CMR. The CMR process can be made simpler with the help of AI solutions that have been proposed to aid in image acquisition, reconstruction, and quality improvement. CMR is usually requested in case of diagnostic suspicion of structural heart disease, and it plays a fundamental role in athletes, especially when echocardiography is normal. Only afterwards it would be appropriate to mention the challenges of image interpretation and the potential role of AI in improving accuracy and efficiency (32, 33).

By strengthening risk assessment, preventing cardiac abnormalities early, and improving athlete treatment planning and monitoring, the incorporation of AI technologies holds the potential to completely transform sports cardiology. A promising avenue for converting conventional screening procedures into more sophisticated, accurate, and predictive medical interventions is provided by this innovation (34, 35). With a focus on their potential to improve PPS and lower the risk of SCD in athletes, Table 1 lists AI applications in sports cardiology.

**Table 1:** Ai applications in sports cardiology.

Screening techniques	New AI tools
Clinical Examination	Analytical Data Collection - Chatbots with AI capabilities make it easier to gather medical history by expediting the procedure and spotting important information that might be overlooked during traditional clinical visits (28).
	Digital Stethoscopes- The identification of various cardiac murmurs in athletes is enhanced by AI enhanced stethoscopes integrated with machine learning algorithms (29).
ECG	AI models offer better detection of CV conditions associated with SCD, increased accuracy in interpreting ECG data, and the capacity to forecast clinical outcomes (30).
	Modern AI algorithms improve the diagnostic precision of ongoing ECG monitoring and exercise stress tests, especially when it comes to detecting heart rhythm abnormalities and coronary artery disease (31).
	Heart rate variability- Power spectral analysis of beat to beat ECG data (32).
	By extending wearable technology's capabilities through AI integration, athletes can benefit from improved risk assessment and continuous cardiac monitoring (33).
Multi-modal Imaging	Clinical professionals can distinguish between pathological conditions and physiological cardiac adaptations with the use of AI-based tools that analyse echocardiographic patterns (34).
	A thorough echocardiographic evaluation of athletes can effectively assess almost all structures and conditions using deep learning techniques. This includes many potentially harmful CV conditions, including congenital anomalies, cardiomyopathy, valvular diseases, aortic root diseases (35).
	Cardiac magnetic resonance (36).
Genomic data analysis	By identifying genetic markers and disease subtypes using AI models on massive genomic datasets, more precise risk stratification and individualised treatment plans are made possible (37).

ECG-Electrocardiography, AI-Artificial Intelligence, CV Cardiovascular, SCD-sudden cardiac death

## Conclusion

Sports cardiology places a lot of emphasis on athlete screening, but there are still a number of issues and problems that need to be resolved in order to enhance the early detection of people who are at risk of sudden cardiac death. In this context, creating required prospective registries that systematically report all cases of SCD in athletes is essential. AI and digital technology integration in sports medicine may improve athletes' assessments, but since this field is developing, strong protocol frameworks will be essential to maximising the potential of these technologies.
